# Controlled Bio-Orthogonal Catalysis Using Nanozyme–Protein Complexes via Modulation of Electrostatic Interactions

**DOI:** 10.3390/ma17071507

**Published:** 2024-03-26

**Authors:** Liang Liu, Xianzhi Zhang, Stefano Fedeli, Yagiz Anil Cicek, William Ndugire, Vincent M. Rotello

**Affiliations:** Department of Chemistry, University of Massachusetts Amherst, 710 N. Pleasant St., Amherst, MA 01003, USA; lliu5@umass.edu (L.L.); xianzhizhang@umass.edu (X.Z.); sfedeli@umass.edu (S.F.); ycicek@umass.edu (Y.A.C.);

**Keywords:** stimuli-responsive catalysis, bio-orthogonal, nanozymes, supramolecular interactions, dielectric screening

## Abstract

Bio-orthogonal chemistry provides a powerful tool for drug delivery systems due to its ability to generate therapeutic agents in situ, minimizing off-target effects. Bio-orthogonal transition metal catalysts (TMCs) with stimuli-responsive properties offer possibilities for controllable catalysis due to their spatial-, temporal-, and dosage-controllable properties. In this paper, we fabricated a stimuli-responsive bio-orthogonal catalysis system based on an enhanced green fluorescent protein (EGFP)–nanozyme (NZ) complex (EGFP-NZ). Regulation of the catalytic properties of the EGFP-NZ complex was directly achieved by modulating the ionic strength of the solution. The dielectric screening introduced by salt ions allows the dissociation of the EGFP-NZ complex, increasing the access of substrate to the active site of the NZs and concomitantly increasing nanozyme activity. The change in catalytic rate of the NZ/EGFP = 1:1 complex was positively correlated with salt concentration from 0 mM to 150 mM.

## 1. Introduction

Controllable catalysis becomes feasible with the development of stimuli-responsive systems that can recognize the microenvironment and respond dynamically [1,2]. Stimuli commonly used are divided into external (temperature, magnetic field, ultrasound, light, or electric pulses) and internal (changes in pH, ionic strength, enzyme concentration, and redox gradients) [3,4,5].

Stimuli-responsive catalysis mediated by bio-orthogonal chemistry is particularly interesting due to its ability to generate therapeutic agents in situ, potentially minimizing off-target effects [6]. Bio-orthogonal chemistry uses abiotic reactions that can occur inside living systems without interfering with native biochemical processes [7,8]. Transition metal catalysts (TMCs) are commonly used catalysts for bio-orthogonal reactions as they can catalyze a wide range of reactions with high catalytic efficiency and bio-orthogonal selectivity [9,10]. However, the hydrophobicity and instability of TMCs in biological environments can make their direct use difficult [11]. TMCs can be loaded into nanomaterials to produce “nanozymes” (NZs) with enhanced stability, biocompatibility, solubility, and catalytic capabilities [12,13,14]. Gold-nanoparticle-based nanozymes offer distinct advantages, including enhanced catalytic efficiency, stability under physiological conditions, and the ability to be easily functionalized for targeted applications, thereby providing a versatile platform for biomedical and environmental applications. The NZs allow therapeutics and imaging agents to be activated in situ from inactive precursors, providing on-demand “drug factories” for several applications, including anticancer [15,16,17,18,19], antimicrobial [20,21], and anti-inflammatory treatments [22].

Nanozymes can potentially be engineered to respond to external or internal stimuli, resulting in controlled catalytic performance [23]. Stimuli-responsive bio-orthogonal nanozymes, designed to react to environmental changes like temperature, pH, or light, offer significant potential for targeted therapeutic applications by enabling localized drug release, thus minimizing off-target effects. Rotello and coworkers developed nanozymes that are activated internally through the modification of the protein corona on gold nanoparticles (AuNPs). They demonstrated that the presence of proteases, by changing the composition of the corona, affects both the activity of the nanozymes and their susceptibility to proteolytic degradation [10]. pH changes in biological environments guide the design of systems for targeted drug delivery, as demonstrated by AuNPs functionalized with pH-responsive ligands for enhanced cellular uptake in acidic conditions [24]. Host–guest interactions, utilized by the Rotello group, enable the control of nanozyme activity through allosteric regulation, with specific molecules acting as gates that can be removed by competitive guests to restore activity [23]. Light-responsive systems, developed by Qu and colleagues, leverage light-gated host–guest interactions for reversible modulation of catalytic activity, allowing for precise control over therapeutic molecule release [25]. Temperature-responsive nanozymes, created by embedding supramolecular assemblies within gold nanoparticle (AuNP) monolayers, exemplify temperature’s role as an external stimulus to activate catalytic functions [21]. These approaches underscore the adaptability and promise of stimuli-responsive nanozymes for use in biomedical fields.

**Figure 1:**
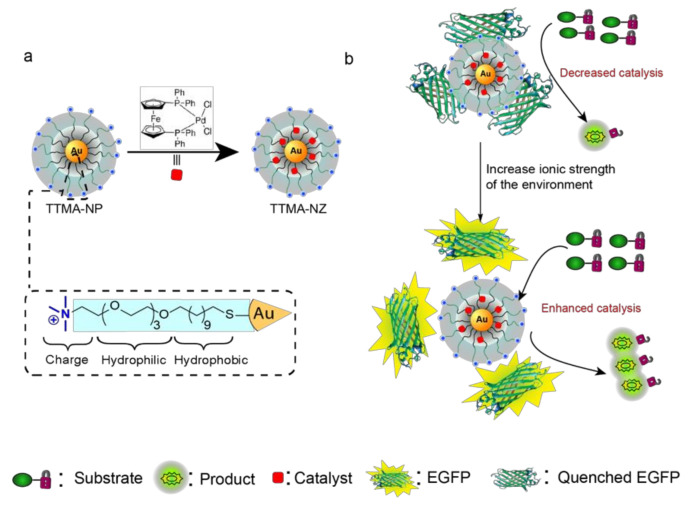
(**a**) Structure of nanoparticles and nanozymes, TTMA-NP and TTMA-NZ. (**b**) Schematic diagram of stimuli-responsive EGFP-NZ complex. Fluorescence can provide an effective readout to quantify interactions between fluorescent proteins and nanoparticles. The catalytic rate of the complex is controlled by ionic strength.

We hypothesized that complexes formed between charged nanozymes and proteins could have potential environmental responsiveness due to their reversible supramolecular interactions [26,27]. In this paper, we present a stimuli-responsive bio-orthogonal catalytic system based on an EGFP-NZ complex, which represents the new formulation. The catalytic properties can be modulated directly by the change in environmental ionic strength (Figure 1). The inhibition mechanism of EGFPs on the catalytic ability of TTMA-NZ is non-competitive inhibition (allosteric inhibition), as indicated in the Lineweaver–Burk plot. We demonstrate that different ionic strengths can regulate the catalytic ability of the EGFP-NZ complex. The competitive electrostatic interaction provided by salt ions allows the dissociation of the EGFP-NZ complex, increasing the access of substrate to the active site of nanozymes. In this way, the catalytic performance can be influenced by manipulating the salt concentration in the environment, affecting the accessibility of the nanozyme active site to the substrate. Based on the results, the NZ/EGFP = 1:1 complex exhibits a positive correlation with salt concentration in terms of catalytic activity.

## 2. Materials and Methods

### 2.1. Materials

All reagents and materials were sourced from Fisher Scientific International, located in Pittsburgh, PA, USA, and Sigma-Aldrich, based in St. Louis, MO, USA, and were used as received, without any additional purification steps. The nuclear magnetic resonance (NMR) spectra for both ^1^H and ^13^C were acquired using a Bruker ADVANCE 400 instrument (Bruker, Billerica, MA, USA). For the analysis of mass spectra via electrospray ionization (ESI-MS), a Bruker MicroTOF-II was employed. The determination of nanoparticle and nanozyme concentrations was conducted following an established methodology that involves measuring the absorbance at a wavelength of 506 nm [28]. Both absorbance and fluorescence readings were taken using a SpectraMax M2 microplate reader from Molecular Devices (San Jose, CA, USA). Transmission electron microscopy (TEM) images were captured by depositing a 10 µL sample of the nanoparticle solution, with a concentration of approximately 5 µM, onto a carbon-coated 400-mesh copper grid, utilizing a JEOL CX-100 electron microscope for imaging (JEOL, Tokyo, Japan). The dynamic light scattering (DLS) measurements for nanoparticles, at a concentration around 1 µM, were performed on a Malvern Zetasizer Nano ZS instrument (Malvern Panalytical, Malvern, UK), using a backscatter angle of 173°.

### 2.2. Synthesis of Ligands

The synthesis route is shown in Supplementary Figure S1. Compound **1** was synthesized by following the previous procedure [29]. Initially, Compound **1**, weighing 200 mg and corresponding to 0.300 mmol (equating to 1 equivalent), was dissolved in 3 mL of anhydrous dichloromethane (DCM). To this solution, 400 µL of trifluoroacetic acid (TFA), which amounts to 5.22 mmol or 17.4 equivalents, was introduced gradually. Following this, 92 µL of triisopropylsilane (TIPS), equating to 71.1 mmol or 1.5 equivalents, was added to the mixture. The reaction was then conducted at ambient temperature under a nitrogen atmosphere, being stirred continuously for 5 h. Subsequent to the reaction, the solvents DCM, TFA, and TIPS were removed via evaporation under reduced pressure. The resulting oil-like residue underwent purification through sequential washes with hexanes (thrice) and dimethyl ether (thrice), followed by drying under high vacuum to yield the TTMA ligand.

### 2.3. Synthesis of the Gold Core

The synthesis of the gold core was achieved using the Brust–Schiffrin two-phase method [30]. This process began with dissolving 1 g of HAuCl_4_ in a mixture composed of 150 mL of MilliQ water and 150 mL of toluene. In a separate step, 2.1 g of tetraoctylammonium bromide (TOAB) was dissolved in a portion of the water–toluene mixture and then added to the main solution under vigorous stirring. To this, pentanediol was gradually introduced into the toluene until the mixture turned white, requiring approximately 0.7 mL. Subsequently, 2.0 g of sodium borohydride was dissolved in 8 mL of water and immediately added to the white mixture. The solution was stirred continuously for 5 h. Following this period, the organic layer was separated and dried under reduced pressure at room temperature. The gold core was then dispersed in hexane and subjected to repeated washing with acetonitrile to completely remove the TOAB. Finally, the organic solvents were evaporated under reduced pressure, resulting in the gold core appearing as a black solid.

### 2.4. Synthesis of TTMA-NP

The process began with the dissolution of a 30 mg gold core in 4 mL of DCM under an argon atmosphere. To this solution, 90 mg of TTMA ligand in a 4 mL DCM/MeOH mixture (1:1 ratio) was gradually added, and the mixture was stirred under nitrogen at room temperature for 72 h. Following this, the organic solvents were removed under reduced pressure. The TTMA-NP was then washed three times with hexanes and three times with a hexanes/DCM mixture (1:1 ratio). Afterwards, it was dispersed in Milli-Q water, dialyzed using a skin membrane with a 10,000 MWCO for 3 days, filtered through a 0.25 μM PES membrane, and concentrated using molecular cut-off filtration (10,000 MWCO) five times.

### 2.5. Encapsulation of Pd into the Monolayer of Nanoparticles

Pd catalyst was encapsulated into TTMA-NP by nanoprecipitation [16]. The catalyst [Pd(dppf)Cl_2_] (5.0 mg) was dissolved in 1 mL of THF/acetone = 1:1, and the AuNPs (20 μM, 0.5 mL) were diluted with DI water to a final concentration of 5 μM (1 mL). The catalyst and AuNP solutions were then mixed, and the organic solvent was gently evaporated. Through this process, the hydrophobic catalyst is enclosed in the particle monolayer to produce NP_Pd. Excess catalysts were removed by dialysis (Snakeskin dialysis tubing, 10 K) against water (5 L) for 24 h and filtering (Millex-GP filter; 25 mm PES, particle size: 0.22 μm). Finally, the nanozymes were purified by ultrafiltration (Amicon Ultra 4, 10 K, 5 × 4000 rpm).

### 2.6. Protein Expression and Purification

EGFPs were expressed in E. coli BL21 DE3 PlysS strain (Millipore, Temecula, CA, USA). Protein expression was carried out in 2 × YT media, induced with 1 mM IPTG, and incubated for 16 h at 18 °C. Cells were then harvested, and the pellets were lysed using 1% Triton-X-100 (30 min, 37 °C)/DNase-I (New England Biolabs, Ipswich, MA, USA) treatment for 20 min. Digested pellets were centrifuged in polypropylene tubes (Fisher) at 14,000 RPM for 30 min. The supernatant was then collected. EGFPs were then purified using HisPur Cobalt columns. After elution, protein was dialyzed twice over 18 h in 1× PBS. Protein purity was confirmed using 12% SDS-PAGE gel. GFP concentration was determined using absorbance at 488 nm.

### 2.7. Fluorescence Titration

Fluorescence titration between TTMA-NZ and EGFPs followed a previously described protocol [31]. Briefly, a change in the fluorescence intensity of EGFPs (50 nM) at 507 nm was measured using an excitation wavelength of 488 nm at various concentrations of TTMA-NZ (0–350 nM) and ionic strength (0, 50, 100, and 150 mM). To create solutions for the salt-dependent interactions between NZs and EGFPs, varying quantities of NaCl were added to the phosphate buffer solution (5 mM, pH 7.4). All experiments were repeated twice and performed in triplicate using a Molecular Devices SpectraMax M2 microplate reader (operating at 37 °C).

### 2.8. Kinetic Study of TTMA-NZ

In a 96-well black plate, TTMA-NZ and pro-Rho were mixed in PBS to obtain a 100 µL solution containing 200 nM TTMA-NZ and 10 µM pro-coumarin (pro-Cou). Pro-Cou-only and TTMA-NZ-only samples were used as negative controls. The kinetic results were obtained by tracking the fluorescence (λex: 375 nm, λem: 446 nm, cutoff: 400 nm) using a Molecular Devices SpectraMax M2 plate reader at 37 °C for 120 min continuously.

## 3. Results and Discussion

### 3.1. Design and Characterization of Nanozymes

**Figure 2:**
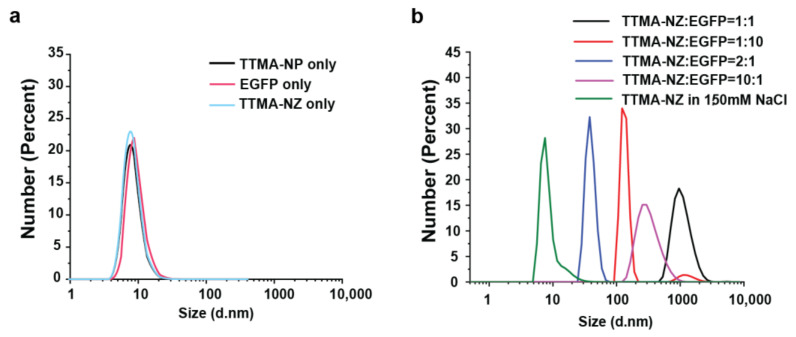
Size distribution of (**a**) TTMA-NP only, EGFPs only, and TTMA-NZ only and (**b**) different ratios of NZ/EGFP and TTMA-NZ only in 150 mM NaCl solution, measured at 5 mM PB, pH = 7.4, 37 °C; each nanoparticle/nanozyme solution was prepared to final concentrations of 0.1 μM.

The ligand design (Figure 1a) consists of three key components designed to maximize catalyst encapsulation and aqueous solubility. The ligands (TTMA) are composed of a hydrophobic linear alkyl chain (C_11_) for stabilizing the nanoparticle and encapsulating the catalyst; a tetra ethylene glycol (TEG) spacer to enhance hydrophobicity and biocompatibility; and a terminal quaternary ammonium (TMA) group to provide NPs with a positive charge. Palladium catalysts were encapsulated inside the monolayer of AuNPs using nanoprecipitation to generate nanozymes (TTMA-NZ). We selected the palladium ferrocene catalyst Pd(dppf)Cl_2_ ([1,1′-bis (diphenylphosphino) ferrocene] dichloropalladium (II) (Figure 1a) due to its efficient catalytic ability [32]. Transmission electron microscopy (TEM) (Figure S4) and dynamic light scattering (DLS) (Figure 2a) of TTMA-NP and TTMA-NZ indicated no aggregation after catalyst encapsulation. The DLS result confirms that TTMA-NZ is structurally stable in the presence of 150 mM NaCl (Figure 2b).

### 3.2. Complexation of Nanozymes by EGFPs

Anionic EGFPs were mixed with cationic TTMA-NZ, forming assemblies through electrostatic interactions. The resulting zeta potential measurements can be found in Figure S5. Ref. [9] Free EGFPs showed a similar size with TTMA-NZ (~10 nm), indicated by DLS (Figure 2a). The formation of the protein complex on nanozymes was validated by the increase in size (Figure 2b). Different ratios of NZ to EGFP result in different assemblies, and there is no clear relationship between the size and ratio of EGFP to NZ, presumably due to differential aggregation.

### 3.3. EGFP-NZ Complex Binding Affinity

**Figure 3:**
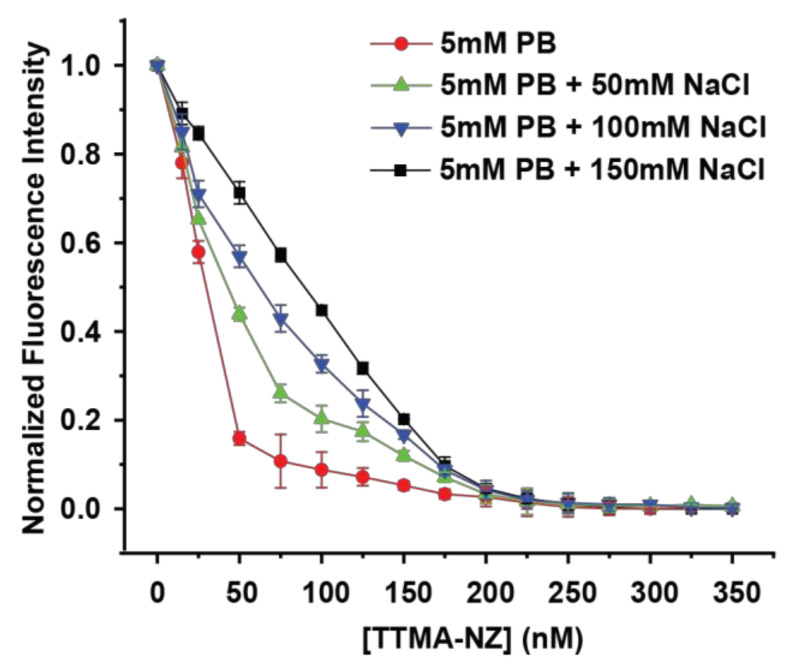
Comparison of the fluorescence titrations between NZs and EGFPs (50 nM) in 5 mM PB containing different NaCl concentrations (0, 50, 100, and 150 mM) (pH 7.4) at 37 °C.

We performed fluorescence titration at different salt concentrations to explore the effect of ionic strength on EGFP-NZ complex binding affinity. Gold nanoparticles strongly quench nearby fluorophores through efficient energy transfer to the gold core [33]. This fluorescence quenching was used to quantify the affinity between the EGFP guests and NZ hosts [34]. Fluorescence titrations (Figure 3) demonstrated that the interaction between EGFPs and NZs provides nanomolar binding affinity at all the ionic strengths tested. A wide range of ionic strengths (0, 50, 100, and 150 mM NaCl) were tested when NZs were titrated with EGFPs. As the concentration of NZs increased, the fluorescence intensity of the protein was gradually quenched. Through nonlinear least-squares curve fitting, we obtained the complex binding constants (K_b_) [31]. Notably, ionic strength cannot reverse the binding between EGFPs and NZs when the ratio of NZ/EGFP = 4:1. The results illustrated that ionic strength has the greatest impact on the dissociation of NZs and EGFPs when NZ/EGFP = 1:1. When the NaCl is absent, the fluorescence of EGFPs in the NZ/EGFP = 1:1 complex was quenched by more than 80%, but when the NaCl concentration is 150 mM, the fluorescence was only quenched by 30%.

The quantitative binding constant study between NZs and EGFPs (Table 1) showed that the K_b_ values decreased monotonically with increasing ionic strength. In the absence of NaCl, the binding strength between NZs and EGFPs was relatively high (K_b_ = 17.8 ± 5.0 × 10^9^ M^−1^), but when the salt concentration reached 50 mM, the affinity between NZs and EGFPs was weakened ~5-fold; then, as the salt concentration increased to 100 mM, K_b_ had a ~20-fold decrease. When the salt concentration reaches the physiological condition, the binding affinity was weakened ~35-fold compared to the solution in the absence of salt. Therefore, the binding affinity between EGFPs and NZs decreases as ionic strength increases [35].

### 3.4. Kinetic Behavior of Nanozymes

**Figure 4:**
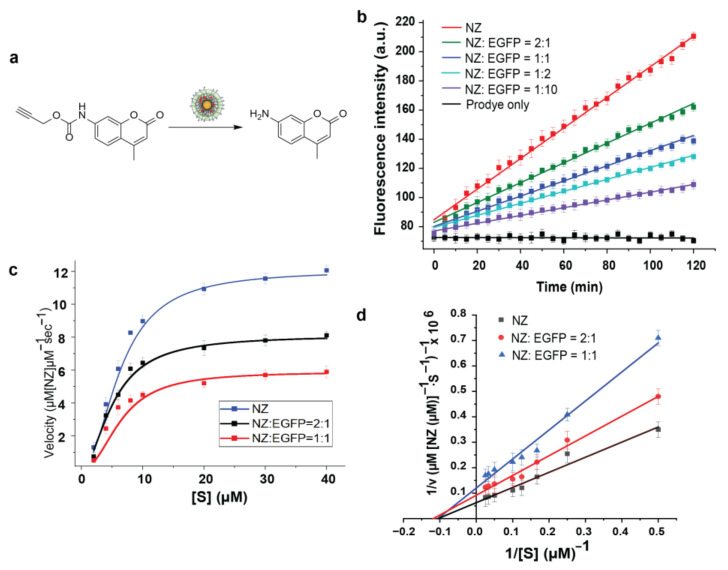
(**a**) Depropargylation of propargylated 7-amino-4-methylcoumarin (excitation: 341 nm; emission: 441 nm) obtained after catalysis. (**b**) An analysis of the kinetics of nanozymes (500 nM) and various complexes of EGFP-NZ converting pro-coumarin (10 µM) into fluorescent products in phosphate-buffered solution (PB, pH 7.4) at 37 °C. Three independent replicates were used to measure the average fluorescence. (**c**) The kinetic curve relating substrate concentration to NZs and the EGFP-NZ complex is illustrated; the line on the graph represents the regression curve as described by Michaelis–Menten kinetics. (**d**) Lineweaver–Burk plot showing non-competitive inhibition of EGFPs to NZs. Based on the kinetic studies conducted in potassium phosphate buffer (5 mM, pH 7.4), EGFPs inhibit catalyst activity through a non-competitive mechanism. Numbers are calculated on a per particle basis. Kinetic studies have been repeated three times for every nanozyme. The error bars indicate standard deviations.

The catalytic activity of palladium-based depropargylation was determined by the activation of the non-fluorescent pro-dye in phosphate buffer (PB, pH = 7.4). Coumarin was chosen due to it having less overlap with the excitation and emission spectra of EGFPs [36,37]. Pro-coumarin was synthesized by propargylation of the coumarin amine using propargyl chloroformate (Synthesis in Supplementary Materials). The fluorescence of coumarin is restored by the catalytic uncaging of the alkyne group mediated by the Pd catalyst (Figure 4a) [22]. In the presence of NZs, linear fluorescence began to increase immediately, while in the absence of NZs, there was no fluorescence change (Figure 4b).

Various ratios of the NZ/EGFP complex were examined to study the catalytic ability of the complex material. As shown in Figure 4b, the catalytic ability of NZs was not eliminated by EGFPs, indicating the complex formed was still catalytically active. However, the catalytic rate of the complex decreased with the increasing amounts of EGFPs. This indicated an inhibition effect of EGFPs on the catalytic ability of NZs. We conducted Michaelis–Menten kinetics and Lineweaver–Burk analysis to understand the inhibition mechanism of EGFPs on NZs. The NZ-EGFP assembly remained stable after catalysis, as indicated by the size distribution measured by DLS, with no significant changes in size observed (see Figure S8).

### 3.5. Inhibition Mechanism of EGFPs on NZs Using Michaelis–Menten Model and Lineweaver–Burk Analysis

Both the NZs and EGFP-NZ complex displayed a classical Michaelis–Menten kinetic model, with the rate of conversion close to the asymptote at high substrate concentrations due to saturation of nanozyme active sites. According to Figure 4c, NZ/EGFP = 1:1 provided a stronger inhibitory effect, exhibiting a lower substrate-saturation maximum rate (Table 2). NZs alone had a larger maximum catalytic rate compared to the EGFP-NZ complex. When NZ/EGFP = 1:1, there was almost a 1-fold decrease in k_cat_. However, the K_M_ of three experiments were almost identical, which shows non-competitive inhibition. We then use Lineweaver–Burk analysis to confirm the inhibition mechanism of EGFPs to NZs (Figure 4d) [38,39]. The result indicated that EGFPs result in non-competitive inhibition (allosteric inhibition) of the catalytic activity. When EGFPs are present, a change in the morphology of NZs occurs, preventing substrates from accessing the active site of the catalyst. A greater amount of EGFPs contribute to the enhancement of this inhibition effect, leading to a lower k_cat_.

### 3.6. Supramolecular Control of Catalysis through Controlling Ionic Strength

**Figure 5:**
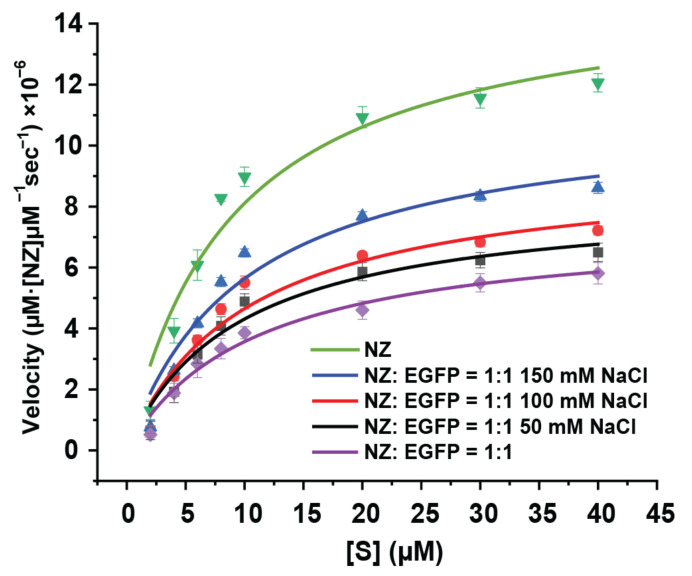
The kinetic curves of NZs and the NZ/EGFP = 1:1 complex under different salt concentrations are shown as a function of substrate concentration; the line on the graph represents the regression curve as described by Michaelis–Menten kinetics.

Due to the non-specific electrostatic interaction between NZs and EGFPs, it is expected that the EGFP-NZ complex will respond to a variety of stimuli like temperature, pH, and ionic strength. In this specific study, we investigated the catalytic rate of NZ/EGFP = 1:1 at different salt concentrations, using NZs as a positive control and NZ/EGFP = 1:1 in the absence of NaCl as a negative control. As shown in Figure 5, all results displayed classical Michaelis–Menten behaviors [40].

According to the parameters obtained by the Michaelis–Menten curve fit (Table 3), the k_cat_ of positive control (NZs in 5 mM PB (pH = 7.4)) was ~2-fold greater than the negative control (NZ/EGFP = 1:1 in 5 mM PB (pH = 7.4)). The catalytic rate of NZ/EGFP = 1:1 was enhanced by 12.2% with an increase in ionic strength from 0 to 50 mM NaCl. With a further increase in salt concentration to normal physiological concentration (150 mM), there was a ~50% increase in k_cat_ relative to the condition without any salt added. According to the above findings, as the ionic strength increases, the catalytic activity has a steady increase due to the dissociation between EGFPs and NZs. The above results showed that our constructed NZ/EGFP = 1:1 complex is an ionic-strength-responsive catalytic material.

## 4. Conclusions

In summary, we have developed a stimuli-responsive nanozyme–protein complex formed by positively charged surface-functionalized gold nanozymes and weakly negatively charged EGFPs. EGFPs act as non-competitive catalytic inhibitors of the nanozymes based on the Lineweaver–Burk analysis, and EGFPs inhibit the catalytic ability of NZs to a greater extent with increasing amounts of EGFPs added. The bio-orthogonal catalytic rate of this complex is responsive to the change in ionic strength. Nanozymes bind to EGFPs via non-specific electrostatic interactions, and salt ions provide dielectric screening that leads to a dissociation of the EGFP-NZ complex, increasing the access of substrate to the active sites of catalysts. Such responsiveness to ionic strength provides the modulation of the catalytic properties of the EGFP-NZ complex material. This study demonstrates a feasible strategy for controllable stimuli-responsive nanozymes. This stimuli-responsive behavior can potentially be used to modulate drug activation for biomedical applications. Additionally, this study establishes a foundation for subsequent research aimed at investigating various stimuli-responsive mechanisms and their potential uses in fields like biosensing, environmental surveillance, and the creation of intelligent materials. Future research might delve into incorporating these nanozyme–protein complexes into broader systems, examining their in vivo behavior and how they interact with diverse biological settings.

## Figures and Tables

**Table 1:** The binding constant values, which are determined based on fluorescence titrations conducted between NZs and EGFPs (the EGFP concentration was kept at 50 nM) [28].

NaCl (mM) in 5 mM PB	0	50	100	150
K_b_ × 10^9^ M^−1^	17.8 ± 5.0	3.5 ± 1.5	0.9 ± 0.2	0.5 ± 0.1

**Table 2:** Kinetic parameters for nanozymes and the EGFP-NZ complex, Mean values ± standard deviation. K_M_, Michaelis constant; k_cat_, catalytic constant; k_cat_/K_M_, second-order rate constant for enzyme-catalyzed reactions.

Parameter	NZ	NZ/EGFP = 2:1	NZ/EGFP = 1:1
K_M_ (µM)	9.0 ± 2.1	9.0 ± 2.0	10.8 ± 1.9
k_cat_ (s^−1^) × 10^−6^	15.3 ± 1.3	10.3 ± 0.8	7.4 ± 0.5
k_cat_/K_M_ (M^−1^ s^−1^) × 10^−1^	18.3 ± 5.8	12.2 ± 3.7	7.2 ± 1.7

**Table 3:** Kinetic parameters for nanozymes and the EGFP-NZ complex, mean values ± standard deviation. K_M_, Michaelis constant; k_cat_, catalytic constant; k_cat_/K_M_, second-order rate constant for enzyme-catalyzed reactions.

	NZ	NZ/EGFP = 1:1			
		150 mM NaCl	100 mM NaCl	50 mM NaCl	0 mM NaCl
K_M_ (µM)	9.0 ± 2.1	9.9 ± 2.4	10.1 ± 2.4	9.3 ± 2.2	10.8 ± 1.9
k_cat_ (s^−1^) × 10^−6^	15.3 ± 1.3	11.2 ± 1.0	9.4 ± 0.8	8.3 ± 0.7	7.4 ± 0.5
k_cat_/K_M_ (M^−1^ s^−1^) × 10^−1^	18.3 ± 5.8	12.3 ± 4.0	10.1 ± 3.2	9.6 ± 3.0	7.2 ± 1.7

## Data Availability

Data are contained within the article.

## Supplementary Materials

The following supporting information can be downloaded at: https://www.mdpi.com/article/10.3390/ma17071507/s1, Figure S1. Synthesis of thioalkyl tetra (ethylene glycol) trimethylammonium (TTMA) ligand; Figure S2. 1HNMR of TTMA ligand; Figure S3. ESI-MS of TTMA ligand; Figure S4. TEM images of (a) TTMA-NP and (b) TTMA-NZ; Figure S5. Zeta potential of (a) TTMA-NZ and (b) EGFP; Figure S6. Synthetic scheme of pro-coumarin; Figure S7. Fitting curve of ionic strength and increase in catalytic rate of EGFP/NZ = 1:1 complex. The black dots represent the percentage increase in Vmax, while the red line represents the linear regression of the data; Figure S8. Size distribution of NZ-EGFP complex before and after catalysis; Figure S9. Absorption and emission spectra of (a) 7-amino-4-methylcoumarin and (b) EGFPs. Ref. [41] can be found in Supplementary Materials.
