# Supplementary material for: Controlled Bio-Orthogonal Catalysis Using Nanozyme–Protein Complexes via Modulation of Electrostatic Interactions
Source: Materials (Basel). 2024 Mar 26;17(7):1507. doi: 10.3390/ma17071507 (PMC11012815; doi:10.3390/ma17071507)
Supplement: Supplementary file 1 [file materials-17-01507-s001.zip › materials-2874516-supplementary.pdf]

materials

Supplementary Materials

Controlled Bio-orthogonal Catalysis using Nanozyme–Protein Complexes via Modulation of Electrostatic Interactions

Liang Liu, Xianzhi Zhang, Stefano Fedeli, Yagiz Anil Cicek, William Ndugire, and Vincent M. Rotello *

Department of Chemistry, University of Massachusetts Amherst, 710 N. Pleasant St., Amherst, MA 01003, USA
* Correspondence: rotello@chem.umass.edu

General information

Unless otherwise noted, all chemical supplies were purchased from Fisher Scientific or Sigma-Aldrich without further purification. ^{1}H NMR was recorded on a Bruker ADVANCE 400 machine. Absorbance and fluorescence data were all collected with a Molecular Devices SpectraMax M2 microplate reader.

Synthesis route of surface ligands and characterization

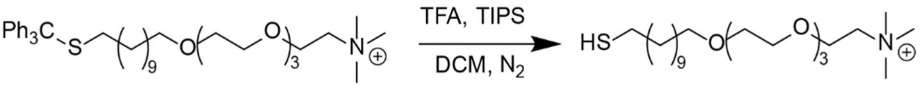

1 TTMA

Figure S1. Synthesis of thioalkyl tetra (ethylene glycol) trimethylammonium (TTMA) ligand.

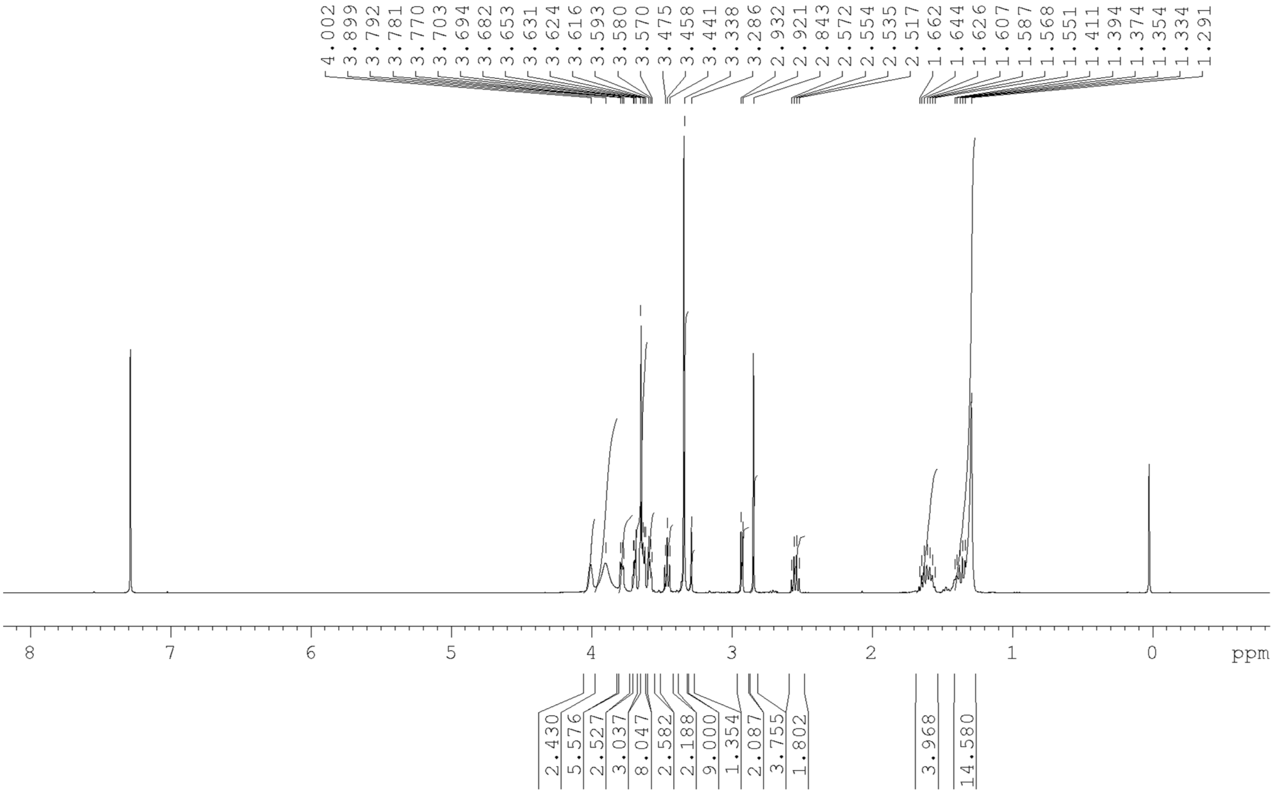

Figure S2. ^{1}HNMR of TTMA ligand.

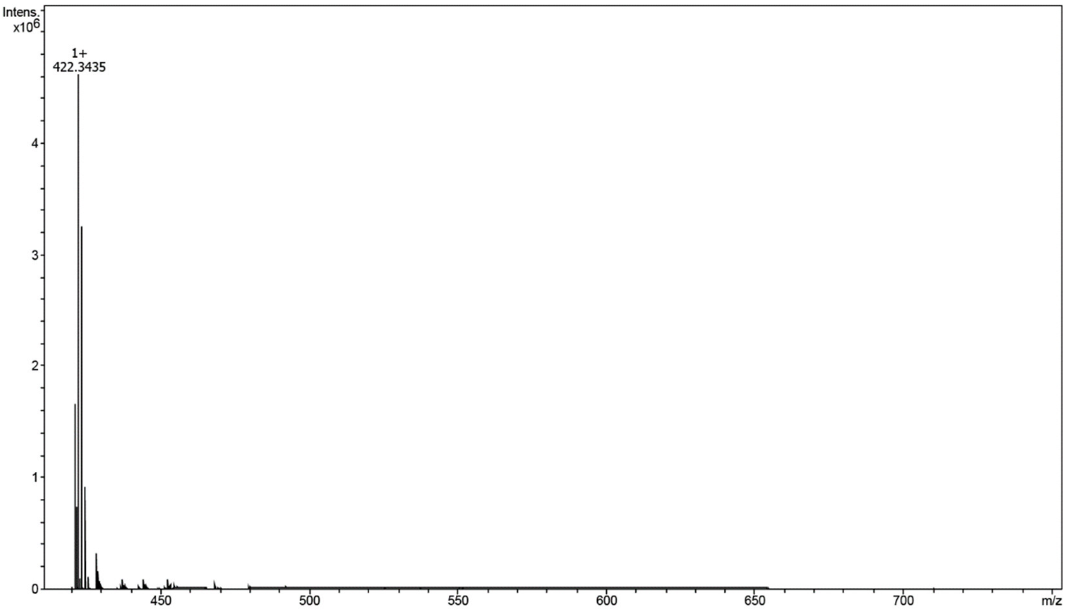

Figure S3. ESI-MS of TTMA ligand.

Characterization AuNP, AuNZ, and EGFP

Malvern Zetasizer Nano ZS equipment with a measurement angle of 173° was used to determine the hydrodynamic diameter of nanoparticles by dynamic light scattering (DLS) in DI water at a concentration of 1 nM (backscatter). The same device was used to test the zeta potential in a 10 mM NaCl solution at a 5 nM concentration. A volume of 10 μL of the required nanoparticle solution were applied on a 300-mesh Cu grid that had a carbon film coating to create the TEM images of the samples. An FEI Tecnai-T12 electron microscope was then used to analyze and photograph the materials.

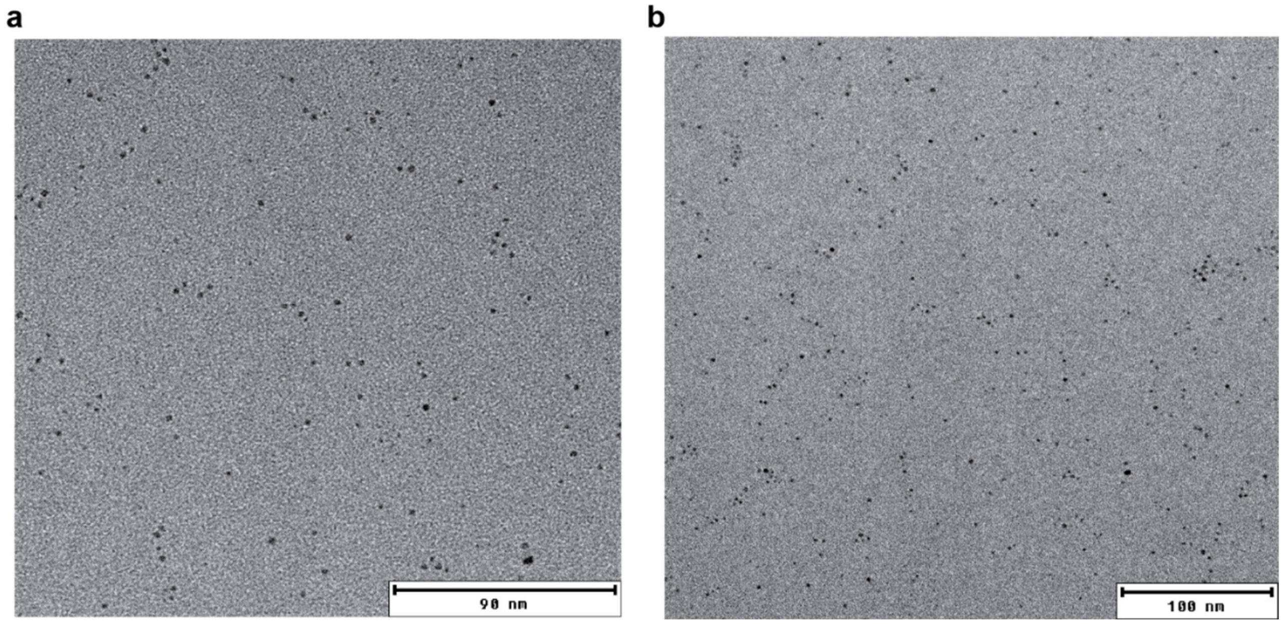

Figure S4. TEM images of (**a**) TTMA-NP and (**b**) TTMA-NZ.

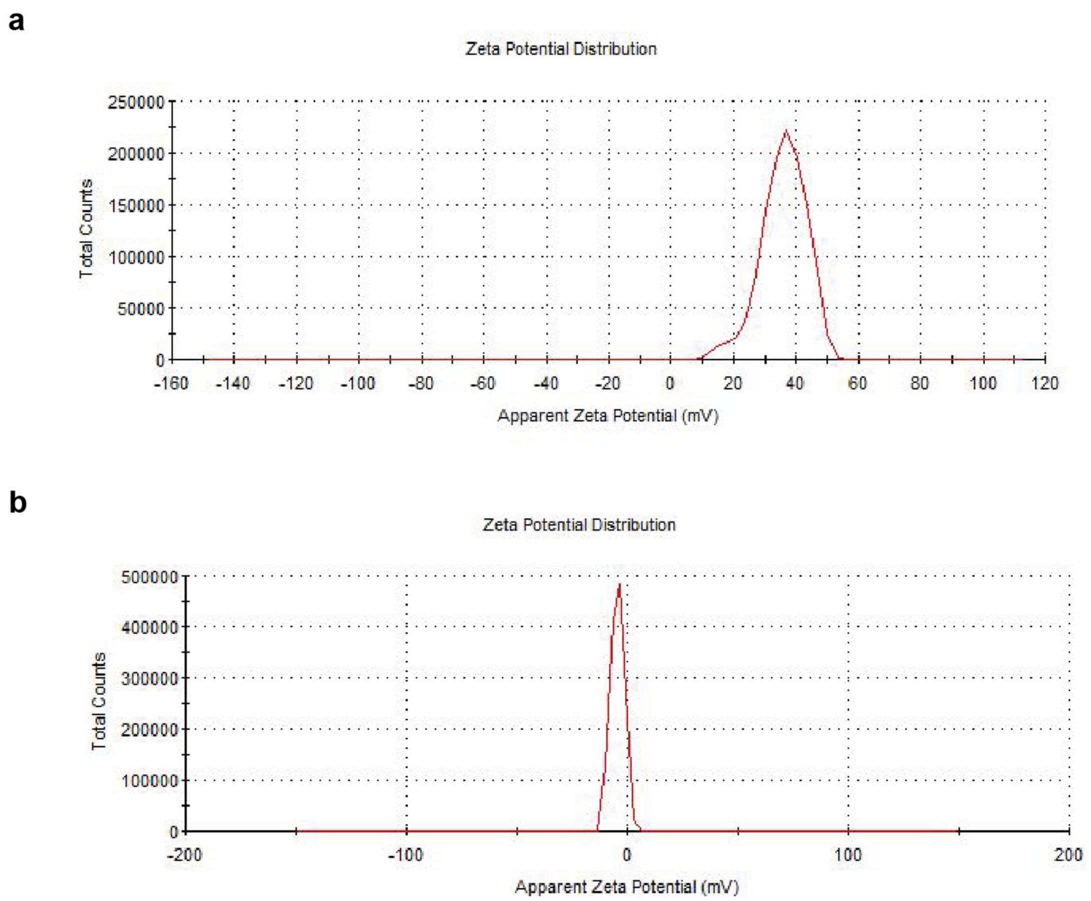

Figure S5. Zeta potential of (**a**) TTMA-NZ and (**b**) EGFP.

Synthesis of pro-coumarin

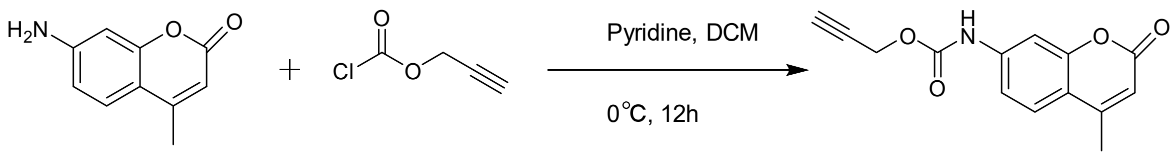

Figure S6. Synthetic scheme of pro-coumarin.

[(4-methyl-2-oxo-2H-chromen-7-yl)oxy]acetonitrile is the protected non-fluorescent pro-coumarin, and the synthesized route is shown above and synthesized based on published procedure. In brief, 300.0 mg of 7-amino-4-methylcoumarin (4) and 151.2 mg of pyridine were dissolved in 10 mL of DCM to create a yellow solution, and 238.5 mg of propargyl carbonyl chloride was then added to the suspension. After stirring at 0 °C for 12 h, the mixture turned into a brilliant yellow suspension. The mixture was then treated with 120 mL of 0.5 M HCl, filtered, and washed with diethyl ether; this procedure was repeated three times to remove excess starting materials, followed by drying to produce 341.5 mg of pro-coumarin. The product was then employed without additional purification (yield: 78%). ^{1}H NMR (500 MHz, DMSO) δ 10.34 (s, 1H), 7.71 (d, J = 8.7 Hz, 1H), 7.53 (d, J = 2.0 Hz, 1H), 7.41 (dd, J = 8.7, 2.1 Hz, 1H), 6.24 (d, J = 1.1 Hz, 1H), 4.82 (d, J = 2.4 Hz, 2H), 3.59 (t, J = 2.4 Hz, 1H), 2.39 (d, J = 1.0 Hz, 3H).

Regression relationship between the improvement of catalytic rate of EGFP/NZ = 1:1 complex and the salt concentration

The fitting of the regression curve comes from the data in Figure 5. The results show that there is a linear relationship between the concentration of NaCl and the improvement of the catalytic performance of the EGFP/NZ = 1:1 complex. The fitting function is shown in Figure S4. This linear relationship can be utilized to predict the catalytic rate of the complexes via ionic strength stimulation.

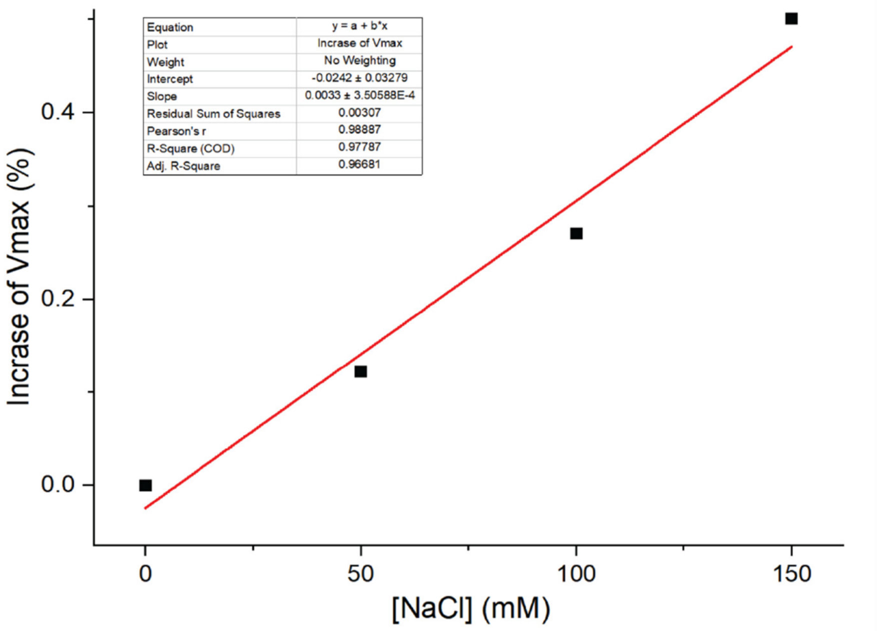

Figure S7. Fitting curve of ionic strength and increase in catalytic rate of EGFP/NZ = 1:1 complex. The black dots represent the percentage increase in Vmax, while the red line represents the linear regression of the data.

Stability of NZ-EGFP complex after catalysis

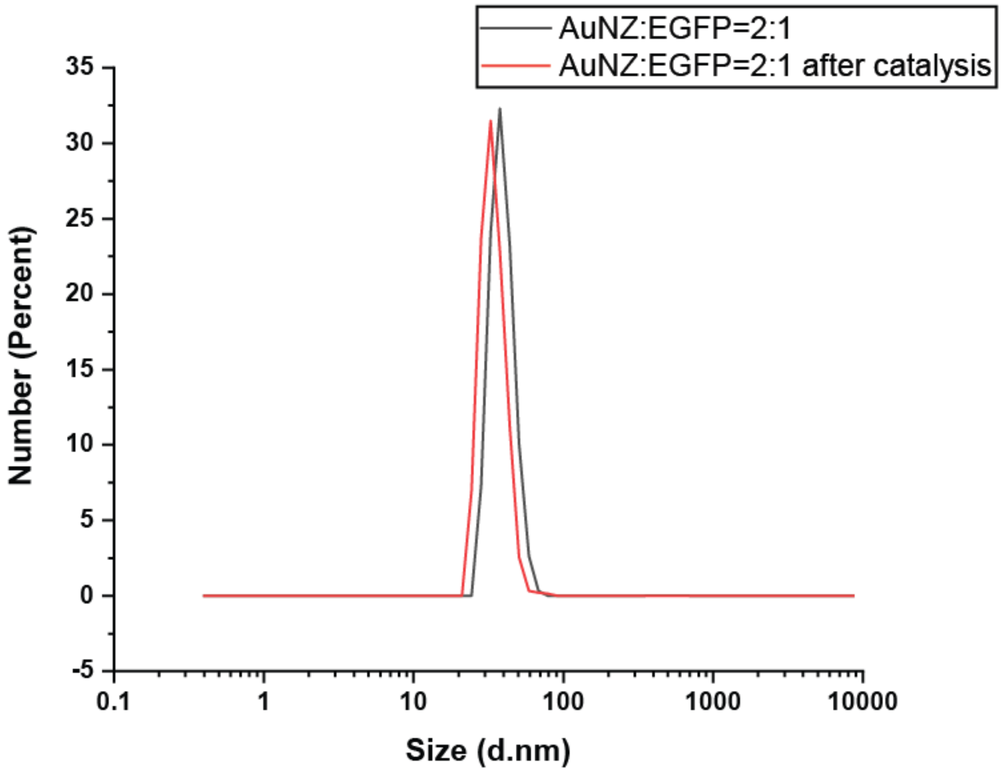

Figure S8. Size distribution of NZ-EGFP complex before and after catalysis.

Spectra of coumarin and EGFPs

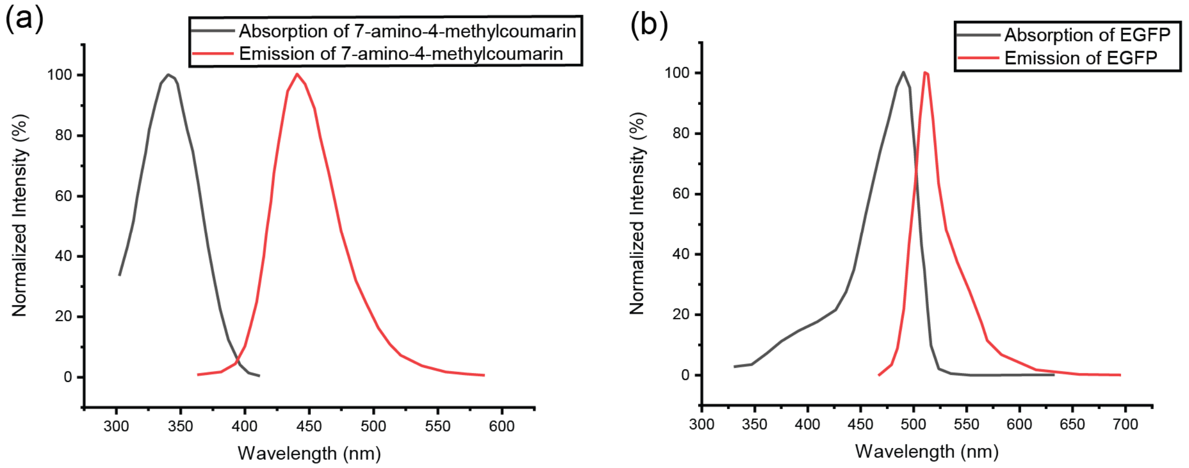

Figure S9. Absorption and emission spectra of (a) 7-amino-4-methylcoumarin and (b) EGFPs.

References

Wang, J.; Cheng, B.; Li, J.; Zhang, Z.; Hong, W.; Chen, X.; Chen, P. R. Chemical Remodeling of Cell-Surface Sialic Acids through a Palladium-Triggered Bioorthogonal Elimination Reaction. *Angew. Chem. Int. Ed Engl.* **2015**, *54*, 5364–5368.
